# Supplementary material for: Comparative genomics provides new insights into the diversity, physiology, and sexuality of the only industrially exploited tremellomycete: Phaffia rhodozyma
Source: BMC Genomics. 2016 Nov 9;17:901. doi: 10.1186/s12864-016-3244-7 (PMC5103461; doi:10.1186/s12864-016-3244-7)
Supplement: Additional file 6: — List of orphan genes with links to PFAM (related to Additional file 1: Table S1). (ZIP 1428 kb) [file 12864_2016_3244_MOESM6_ESM.zip › PFAM_UNIQUE/G05467_P.html]

#                                                                            --- full sequence --- -------------- this domain -------------   hmm coord   ali coord   env coord
  
# target name        accession   tlen query name           accession   qlen   E-value  score  bias   #  of  c-Evalue  i-Evalue  score  bias  from    to  from    to  from    to  acc description of target
  
#------------------- ---------- ----- -------------------- ---------- ----- --------- ------ ----- --- --- --------- --------- ------ ----- ----- ----- ----- ----- ----- ----- ---- ---------------------
  
G05467\_P             -            127 TPR\_12               PF13424.1     78     0.016   13.8   0.0   1   1   0.00022     0.024   13.3   0.0     3    67    52   114    50   118 0.83 - Tetratricopeptide repeat  
G05467\_P             -            127 TPR\_14               PF13428.1     44      0.02   14.2   0.0   1   3       5.8   6.6e+02    0.2   0.0    28    43    19    34    13    47 0.64 - Tetratricopeptide repeat  
G05467\_P             -            127 TPR\_14               PF13428.1     44      0.02   14.2   0.0   2   3      0.75        85    2.9   0.0     3    24    56    77    54    78 0.85 - Tetratricopeptide repeat  
G05467\_P             -            127 TPR\_14               PF13428.1     44      0.02   14.2   0.0   3   3     0.035       3.9    7.1   0.0     5    32    97   124    93   126 0.82 - Tetratricopeptide repeat  
G05467\_P             -            127 TPR\_19               PF14559.1     68    0.0057   15.6   0.0   1   2    0.0086       1.1    8.4   0.0    26    49    55    78    49    93 0.89 - Tetratricopeptide repeat  
G05467\_P             -            127 TPR\_19               PF14559.1     68    0.0057   15.6   0.0   2   2      0.11        13    4.9   0.0    31    57    99   125    93   126 0.90 - Tetratricopeptide repeat  
G05467\_P             -            127 TPR\_21               PF09976.4    145     0.023   13.3   0.1   1   1   7.4e-05      0.04   12.5   0.1    87   139    56   114    48   116 0.82 - Tetratricopeptide repeat
